# Thrombosis in the Surgically Corrected Anomalous Right Coronary Artery after Reimplantation in Aorta

**DOI:** 10.1155/2017/5832692

**Published:** 2017-12-31

**Authors:** Ata Bajwa, Bhanu Gupta, Lina Ya'qoub, Steven B. Laster, Randall Thompson

**Affiliations:** ^1^Mid America Heart Institute, Saint Luke's Hospital and University of Missouri-Kansas City School of Medicine, Kansas City, MO, USA; ^2^University of Kansas Medical Center, Kansas City, KS, USA

## Abstract

A 32-year-old African American female presented with dyspnea, and after several cardiac diagnostic tests, the diagnosis of an anomalous origin of the RCA from the pulmonary trunk was established by multislice coronary CT angiography. She underwent surgical correction with reimplantation of the RCA, from the pulmonary artery to the aortic root. However, 10 days after surgery, she developed frequent episodes of chest pain, and repeat coronary CTA showed a partially occlusive thrombus in the surgically reimplanted RCA. Anticoagulation with warfarin resulted in complete resolution of the patient's symptoms.

## 1. Introduction

Anomalous origin of the right coronary artery (RCA) from the pulmonary artery (ARCAPA) is a rare (0.002%) yet potentially life-threatening condition due to heart failure, coronary ischemia, or sudden cardiac death in infancy, adolescence, or adulthood due to fatal arrhythmia. Various imaging modalities are available to establish the diagnosis of this rare condition. Echocardiography might aid in the diagnosis, but magnetic resonance angiogram (MRA) and multislice-gated coronary CT angiogram are more reliable modalities. Among the various surgically corrected techniques, reimplantation of the anomalous RCA to the aortic root is considered a preferred method. We present a case of recurrent chest pain after thrombosis of the surgically corrected ARCAPA with resolution of symptoms after systemic anticoagulation.

## 2. Case

**Figure 1:**
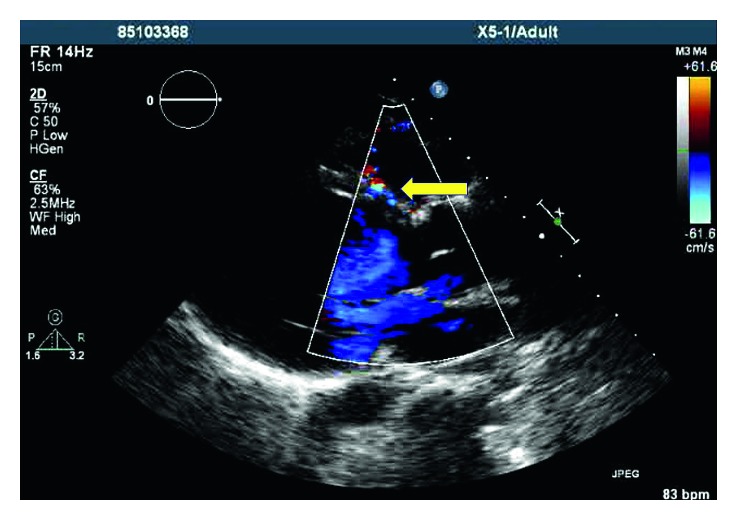
Yellow arrow head pointing at the abnormal linear flow along the interventricular septum, suggestive of dilated left anterior descending artery.

**Figure 2:**
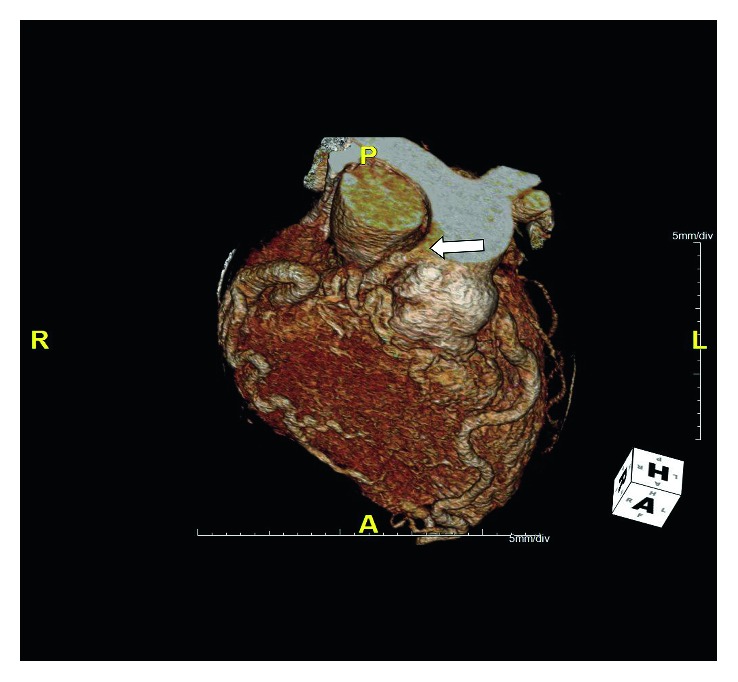
Coronary computed tomography angiogram (volume rendered image) demonstrates the anomalous origin of the right coronary artery (RCA) from the pulmonary trunk (white arrow).

A 32-year-old African American female, mother of seven healthy children, presented to our clinic for evaluation of dyspnea on exertion and chest heaviness of four-year duration. Her clinical exam was remarkable for a continuous murmur along the left sternal border. Echocardiogram was remarkable for an abnormal linear flow along the interventricular septum as shown in Figure 1 and Supplementary Video Clip 1. Coronary angiography demonstrated extremely dilated, diffusely ectatic, and tortuous left coronaries without any separate ostial RCA origin. Despite vigorous injection of X-ray contrast, rapid dilution occurred due to high flow in the left coronary system, as depicted in Supplementary Video Clip 2. On right heart catheterization, there was a step-up of oxygen saturation from 82% in the right ventricle to 87.5% in the pulmonary artery, raising suspicion of left-to-right shunt. The ventricular stroke volume was measured at 113 ml, and cardiac output was high at 11.1 L/min. Multislice-gated coronary CT angiogram revealed an anomalous origin of an extremely dilated and tortuous appearing right coronary artery (RCA) from the pulmonary trunk (Figure 2).

**Figure 3:**
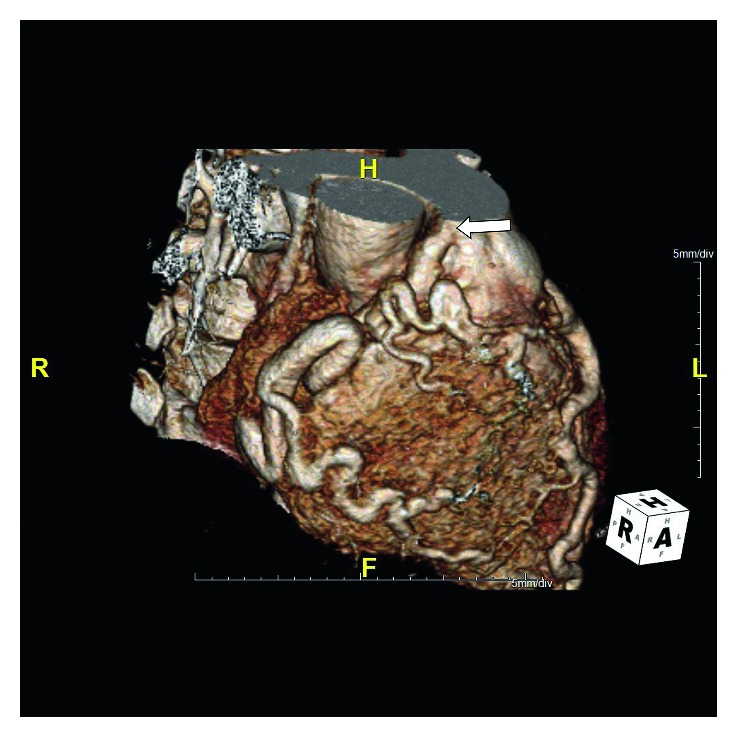
Coronary computed tomography angiogram (volume rendered image) demonstrates another view of the anomalous origin of the right coronary artery (RCA) from the pulmonary trunk (white arrow).

**Figure 4:**
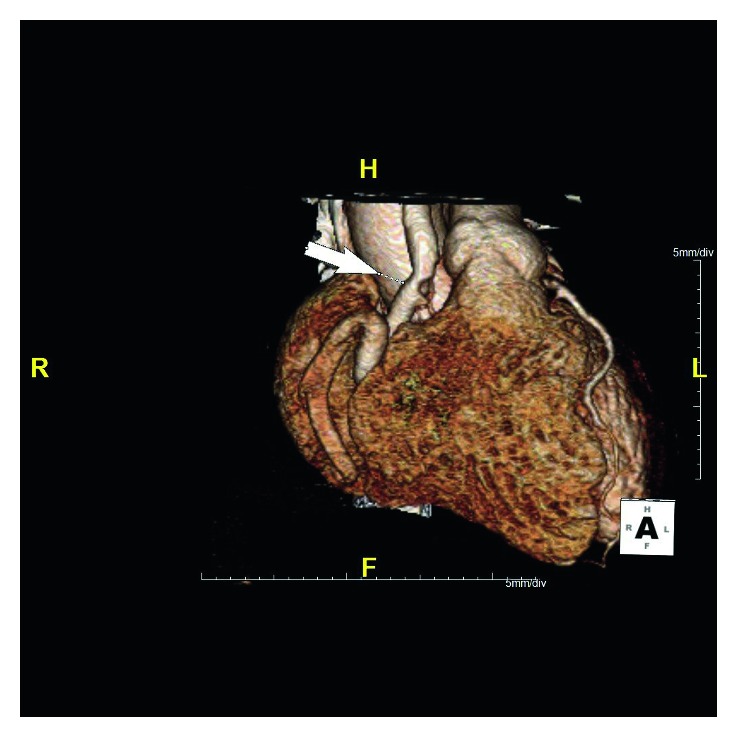
Coronary computed tomography angiogram (volume-rendered image) obtained after her surgery points at the surgically implanted right coronary artery (RCA) in the anterior ascending aorta. There is a filling defect corresponding to a partially occlusive thrombus.

**Figure 5:**
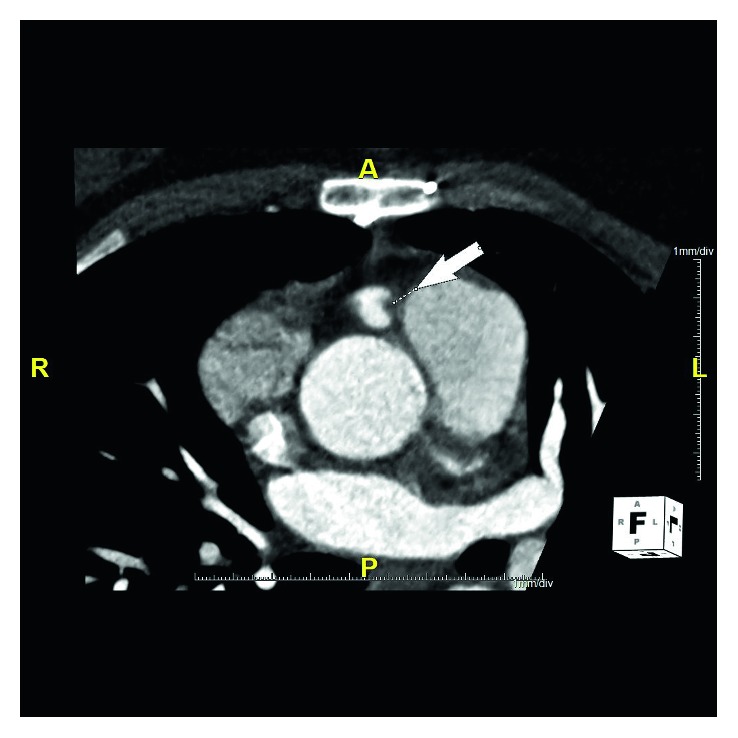
Cross-sectional view of the coronary CTA, after surgery, shows the filling defect in the implanted RCA consistent with partially occlusive thrombus.

**Figure 6:**
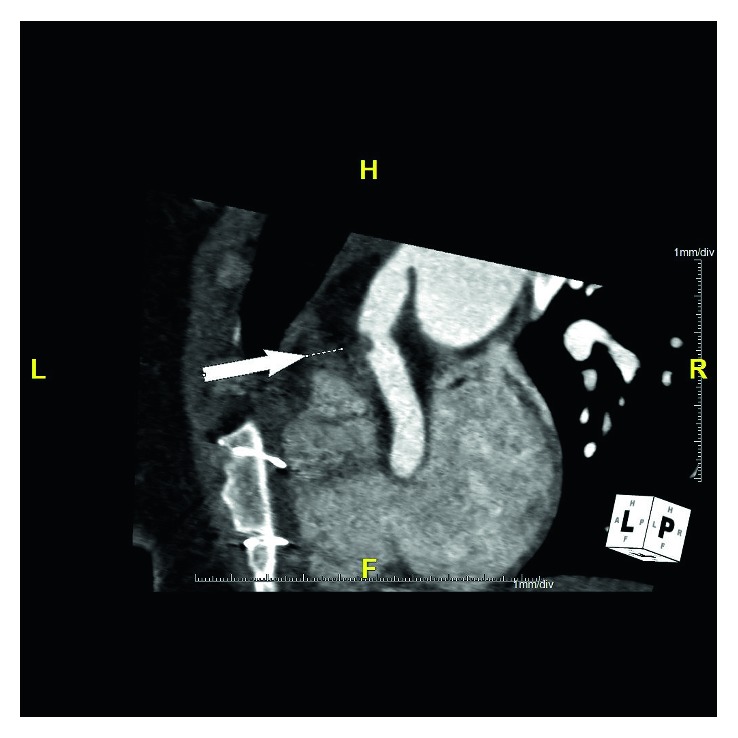
Parasagittal view of the coronary CTA performed after the patient's surgery again demonstrates the partially occlusive thrombus in the implanted RCA as pointed by the arrow.

The patient was offered surgical correction of ARCAPA but continued to defer it despite being symptomatic with frequent clinic visits. Two years later, she decided to schedule her surgery at an outside hospital and underwent reimplantation of the anomalous RCA into the aorta on 14 July 2015. Postoperatively, she initially felt better but developed sharp, substernal chest pain and shortness of breath 10 days later. She continued to have frequent emergency room visits and underwent testing including EKGs, troponins, and CT pulmonary angiogram of the chest, all of which were reported as unremarkable. Subsequently, she underwent a repeat coronary CT angiogram which showed that the RCA was surgically implanted in the anterior ascending aorta (Figures 3 and 4) and had a nonobstructing bulky thrombus in its proximal segment (Figures 5 and 6). She was started on oral anticoagulation therapy with warfarin and continued to follow-up in our clinic with improvement in her symptoms over the course of next few months.

## 3. Discussion

Anomalous origin of the right coronary artery from the pulmonary artery (ARCAPA) is a rare yet potentially life-threatening condition [1]. In adults with ARCAPA, the RCA wall tends to be very thin and fragile and serves as a retrograde venous conduit from the left coronary circulation into the pulmonary artery [1, 2]. This anomalous connection results in a left-to-right shunt that explains both the step-up in oxygen saturation in the pulmonary artery and the high cardiac output seen in these cases, including our case [3]. Anomalous origin of the left coronary artery from the pulmonary artery (ALCAPA) is a sister counterpart of ARCAPA and is relatively more commonly encountered. ALCAPA represents approximately 0.5% of congenital heart diseases compared to 0.002% by ARCAPA. Both of these anomalies can be encountered at any age but ALCAPA tends to become symptomatic early in life and is therefore more frequently diagnosed in infancy and childhood while ARCAPA is usually seen in adolescents and adults [4]. Unlike ARCAPA, physiology of ALCAPA results in significant myocardial ischemia due to inadequate perfusion pressure and low oxygen saturation from the pulmonary artery circulation. The ischemia develops early during infancy in most cases while others may present in early childhood or adulthood albeit with consequences of dilated cardiomyopathy, mitral regurgitation, malignant arrhythmias, and sometimes, even, sudden death [5].

The clinical presentation of ARCAPA varies widely from heart failure and/or coronary ischemia [2, 6, 7]. Cases of sudden cardiac death in otherwise healthy individuals have also been reported, with some cases being described with coexistent coronary atherosclerosis [6, 8]. ARCAPA may be associated with other congenital and acquired heart diseases, including aortopulmonary window, truncus arteriosus, anomalous subclavian artery, tetralogy of Fallot, constrictive pericarditis, bicuspid aortic valve, and mitral regurgitation [7, 9–15].

Advanced cardiac imaging modalities, magnetic resonance angiogram (MRA), or multislice-gated coronary CT angiogram are the definitive diagnostic tests [8]. Multislice-gated coronary CT angiogram has become an increasingly popular test since it is noninvasive and cost-effective and provides accurate anatomical details for surgical correction [7, 8, 16].

Surgical correction of ARCAPA is recommended even in asymptomatic patients with good overall outcomes [1, 3, 10, 12–14]. The most common surgical approach for this condition is to reimplant the anomalous RCA into the correct right aortic sinus. This approach was used in our patient. Another approach is complete ligation of the anomalous RCA proximally with insertion of a saphenous vein conduit from the aorta to the distal RCA, thus restoring normal antegrade coronary flow. “Takeuchi procedure,” occasionally performed in children, is a different surgical option in which an aortopulmonary window is created, and the flow between the anomalous right coronary ostium and the aorta is established using an intrapulmonary baffle or tunnel [15, 17, 18].

Reimplanted coronary arteries appear to be more susceptible to thrombosis. We found two other cases where thrombosis of the corrected anomalous coronary artery was described after reimplantation procedure [19, 20]. Our case is a third example of such complication. The exact mechanism of thrombus formation is not clear, but plausible explanations are change from the high flow to the low flow coronary system after the surgical correction and loss of physiological arterial compliance. It is interesting to note that the thrombus was formed in the proximal part of the implanted anomalous coronary artery in our case as well as the case described by Han and colleagues [19]. Like other thrombotic conditions, warfarin therapy for 3–6 months is used for the treatment of this complication [19]. No data exist regarding the use of newer oral anticoagulant in these situations.

In conclusion, anomalous right coronary artery from pulmonary artery is a rare congenital anomaly. Surgical correction is of low risk with long-term outcomes. However, thrombotic occlusion of reimplanted RCA should be considered in differential diagnosis. CT coronary angiography may be considered to help establish diagnosis in the early postoperative stage.

## Supplementary Materials

Supplementary Materials Video Clip 1: transthoracic echocardiogram. Parasternal long axis view revealed abnormal linear flow along the interventricular septum (yellow arrow). Video Clip 2: coronary angiography with ventriculogram. Left heart catheterization with ventriculogram showed large, diffusely ectatic, and tortuous coronary arteries (left anterior descending (yellow arrow), circumflex artery (red arrow)). Given the extremely dilated coronaries, it was not possible to clearly visualize coronary arteries even after injection of large amount of contrast dye into coronaries.
