# Supplementary material for: Thrombosis in the Surgically Corrected Anomalous Right Coronary Artery after Reimplantation in Aorta
Source: Case Rep Cardiol. 2017 Dec 31;2017:5832692. doi: 10.1155/2017/5832692 (PMC5804374; doi:10.1155/2017/5832692)
Supplement: Supplementary Materials — Video Clip 1: transthoracic echocardiogram. Parasternal long axis view revealed abnormal linear flow along the interventricular septum (yellow arrow). Video Clip 2: coronary angiography with ventriculogram. Left heart catheterization with ventriculogram showed large, diffusely ectatic, and tortuous coronary arteries (left anterior descending (yellow arrow), circumflex artery (red arrow)). Given the extremely dilated coronaries, it was not possible to clearly visualize coronary arteries even after injection of large amount of contrast dye into coronaries. [file 5832692.f1.pptx]

## Slide 1
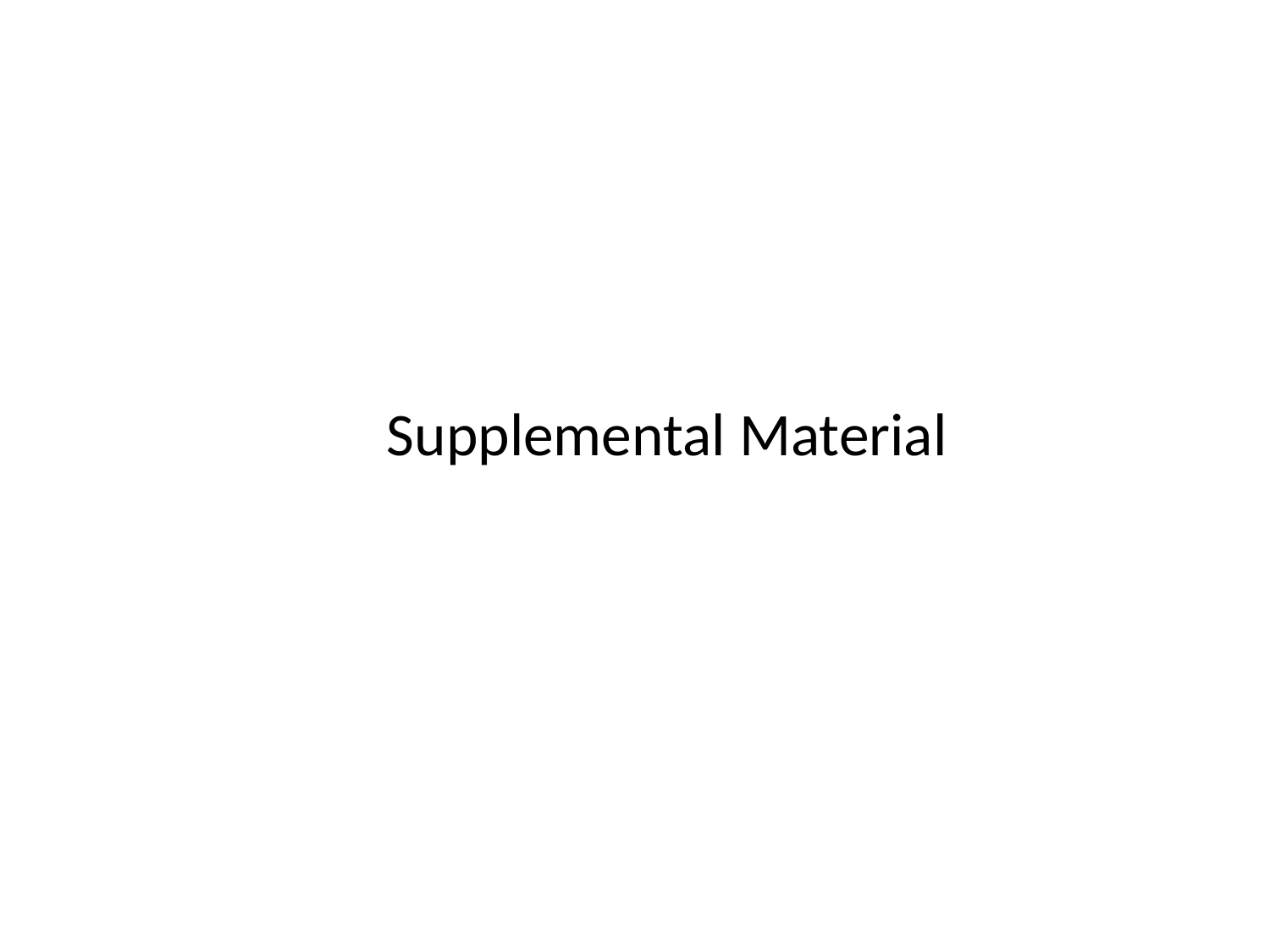

# Supplemental Material

## Slide 2
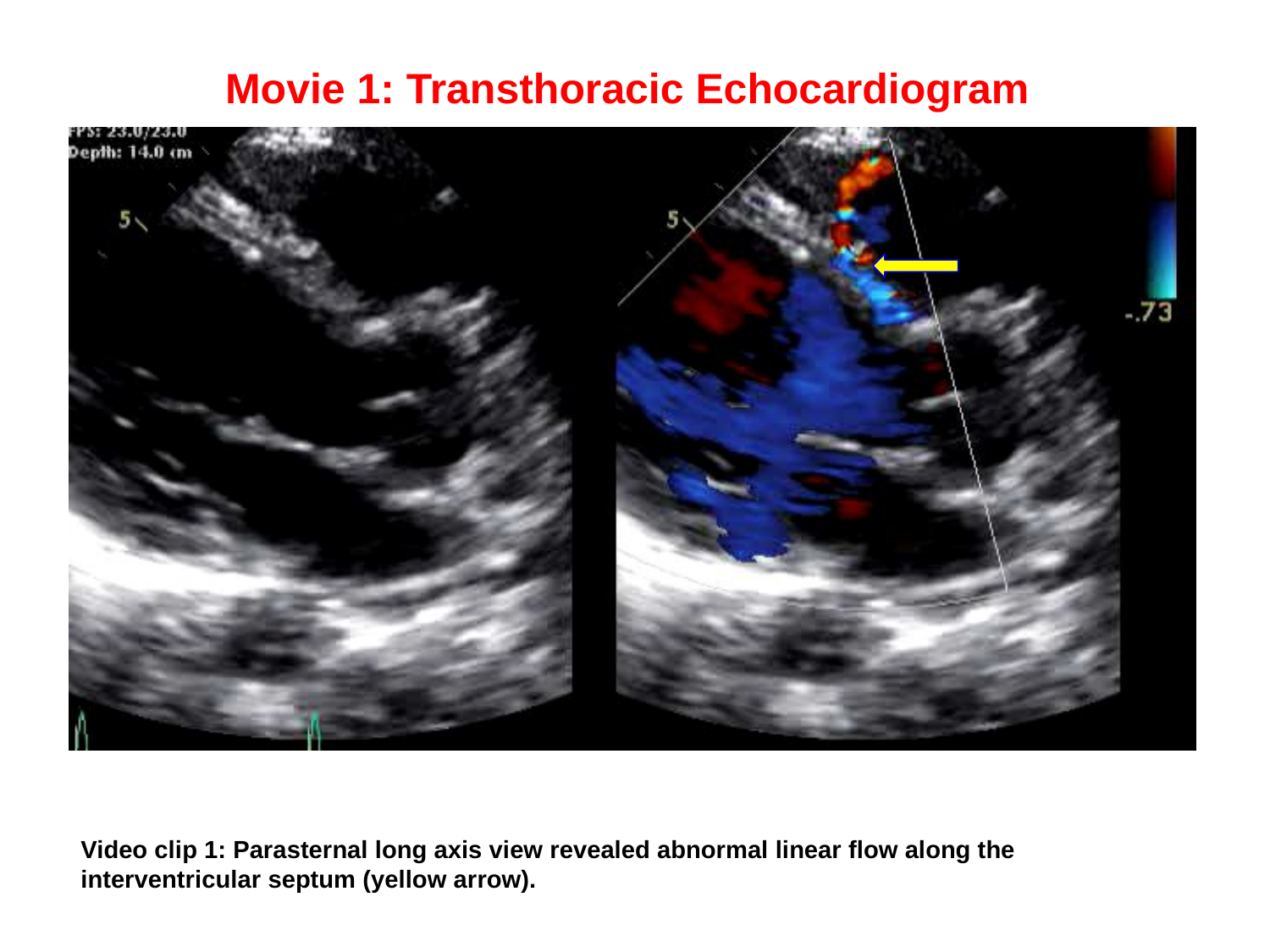

# Movie 1: Transthoracic Echocardiogram
Video clip 1: Parasternal long axis view revealed abnormal linear flow along the interventricular septum (yellow arrow).

## Slide 3
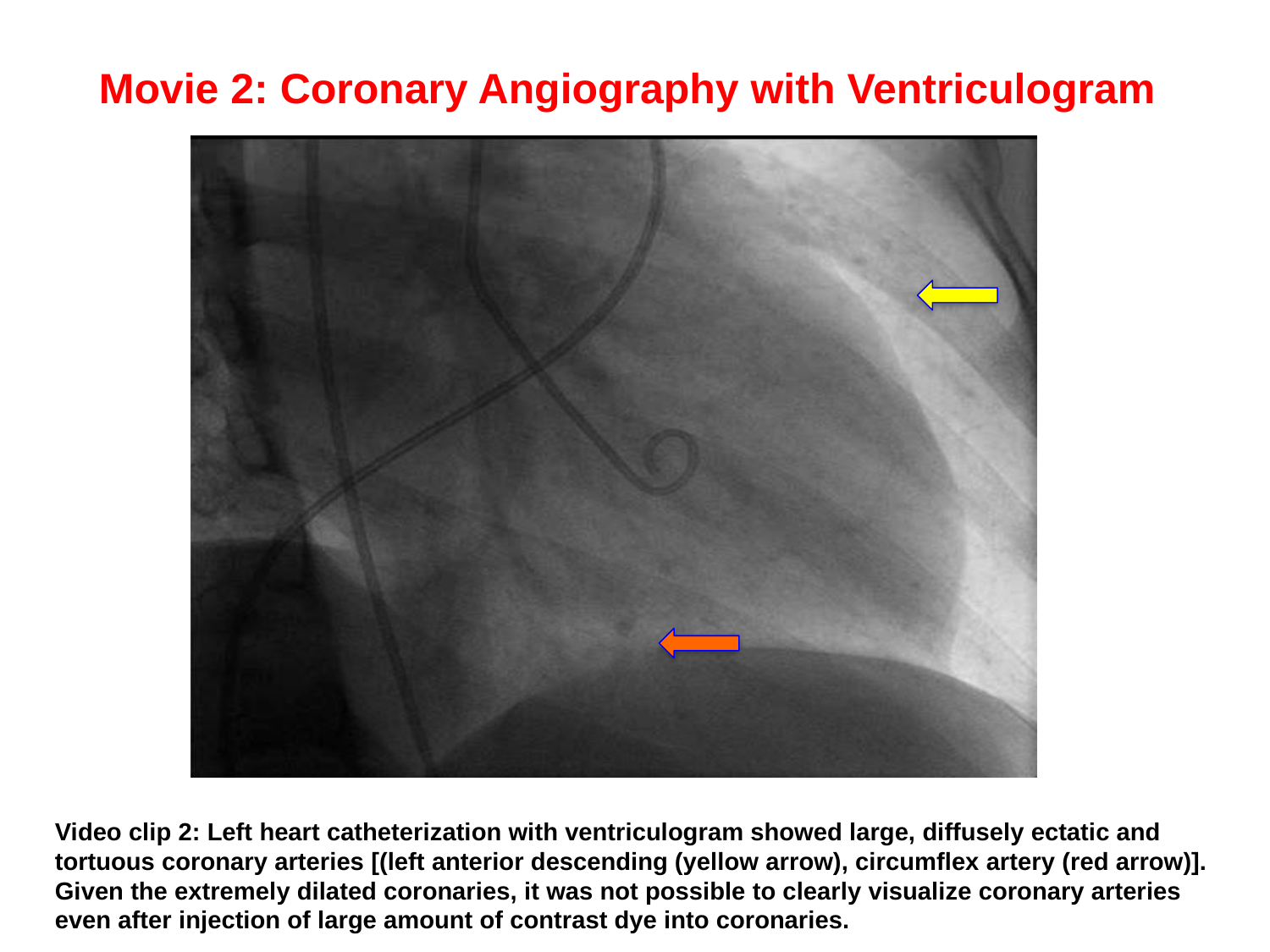

# Movie 2: Coronary Angiography with Ventriculogram
Video clip 2: Left heart catheterization with ventriculogram showed large, diffusely ectatic and tortuous coronary arteries [(left anterior descending (yellow arrow), circumflex artery (red arrow)]. Given the extremely dilated coronaries, it was not possible to clearly visualize coronary arteries even after injection of large amount of contrast dye into coronaries.
